# Case report: administration of immune checkpoint inhibitor for *SMARCB1* (INI1)-negative rhabdoid carcinoma with microsatellite instability (MSI)-high in the right colon

**DOI:** 10.1186/s40792-023-01594-y

**Published:** 2023-02-03

**Authors:** Toshinori Kobayashi, Yuki Matsui, Hisanori Miki, Masahiko Hatta, Mitsuaki Ishida, Hironaga Satake, Mitsugu Sekimoto

**Affiliations:** 1 Department of Surgery, Kansai Medical University, 2-5-1, Shinmachi, Hirakata, Osaka 573-1010 Japan; 2 Department of Pathology, Osaka Medical and Pharmaceutical University, 2-7, Daigaku-Machi, Takatsuki, Osaka 569-8686 Japan; 3 Department of Medical Oncology, Kochi Medical School, Nankoku, 7838505 Japan

**Keywords:** *SMARCB1*, INI1, Rhabdoid carcinoma, MSI-high, Pembrolizumab, Immune checkpoint inhibitors

## Abstract

**Background:**

Malignant tumors with rhabdoid features are rare, highly aggressive, and some of them are characterized by *SMARCB1* (INI1) loss. Although cases of rhabdoid carcinoma are extremely rare, its occurrence in the colon has been reported previously.

**Case presentation:**

A 71-year-old Japanese female patient presented with loss of appetite, fatigue, and weight loss. Computed tomography demonstrated a tumor in the right colon that infiltrated the surrounding kidneys and swelling of the left supraclavicular and periaortic lymph nodes. Laparotomy revealed that the tumor was unresectable because it had directly invaded the head of the pancreas and duodenum. Therefore, ileocecal vascularized bulky lymph nodes were sampled, and gastrojejunostomy with Braun’s anastomosis and ileotransversostomy were performed as palliative procedures. Histopathological examination of the lymph nodes revealed that the neoplastic cells had rich eosinophilic cytoplasm and eccentrically located large nuclei characteristic of rhabdoid carcinoma. In addition, these neoplastic cells lacked *SMARCB1* expression; therefore, the patient was diagnosed with *SMARCB1*-negative rhabdoid carcinoma. The postoperative course was uneventful. Molecular analysis confirmed that the neoplastic cells had high microsatellite instability (MSI); therefore, two cycles of pembrolizumab were administered. However, no clinical benefit was noted, and the patient died 3 months postoperatively.

**Conclusion:**

This is the first report of a case of *SMARCB1*-negative rhabdoid colon carcinoma with high MSI treated with pembrolizumab. Rhabdoid carcinoma is highly aggressive; therefore, additional studies are required to determine the therapeutic strategy for *SMARCB1*-negative rhabdoid colorectal carcinoma.

## Background

Malignant tumors with rhabdoid features are extremely rare. These tumors are characterized by a rhabdomyoblast-like appearance due to the presence of neoplastic cells with cytoplasm containing rich hyaline-like filamentous paranuclear inclusions and eccentrically located large round-to-oval nuclei with conspicuous nucleoli. Sporadic occurrence of this type of tumor has been reported in various organs [1–3]. Prototypes of malignant tumors with rhabdoid features include malignant rhabdoid tumors of the kidneys and atypical teratoid/rhabdoid tumors of the central nervous system. These highly aggressive tumors frequently demonstrate loss of *SMARCB1* (encoding INI1 protein) expression [4, 5]. It was demonstrated that carcinomas with rhabdoid features (rhabdoid carcinomas) show frequent loss of *SMARCB1* and a highly aggressive course [1, 2]. Rhabdoid carcinomas can occur in the gastrointestinal tract [1–3]. Agaimy et al. analyzed the clinicopathological features of 39 previously reported and two new cases of rhabdoid carcinoma. The most common site of this type of tumor was the stomach (13 cases), followed by the colon (11 cases), small intestine (10 cases), and esophagus (5 cases), although not all of the previously reported cases involved the loss of *SMARCB1* [1]. To the best of our knowledge, only five cases of rhabdoid carcinoma with *SMARCB1* loss occurring in the colorectum have been reported [1, 2, 6–8]. Herein, we report the first case of *SMARCB1*-negative rhabdoid right-sided colon cancer treated with immune checkpoint inhibitors (ICIs) because of microsatellite instability (MSI)-high status. Further, we discuss the clinicopathological features of this type of tumor.

## Case presentation

**Fig. 1:**
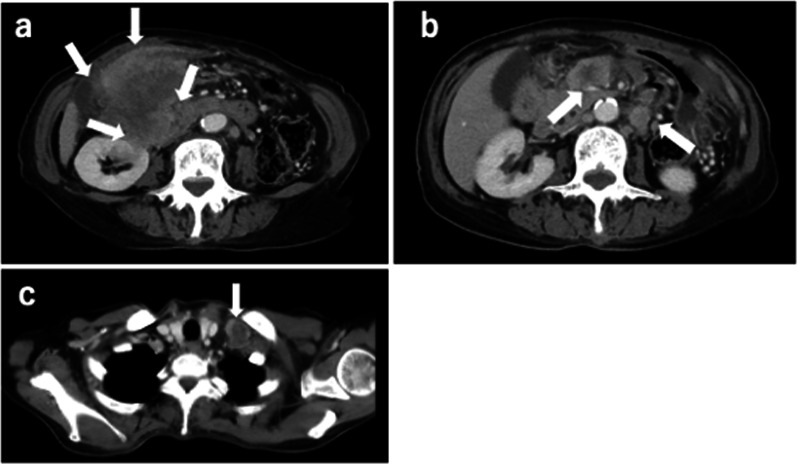
Computed tomography scan. **a** A tumor in the right transverse colon, directly invading the right kidney. **b**, **c** Swelling of the periaortic (**b**) and left supraclavicular lymph nodes (**c**) (arrows)

**Fig. 2:**
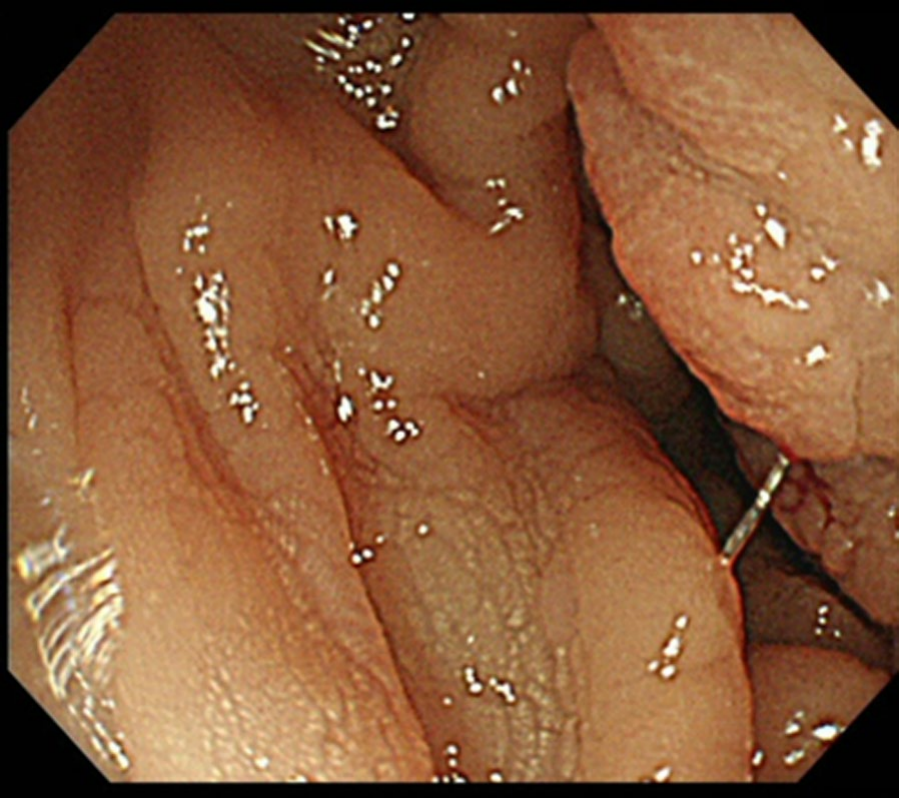
Colonoscopic findings. The presence of edematous mucosa in the right transverse colon is seen, however, no tumorous lesion is observed

A 71-year-old Japanese female patient, with no remarkable clinical history, presented with loss of appetite, fatigability, and weight loss of 8 kg over 3 months when treated at an outpatient clinic. Computed tomography findings demonstrated a tumor, measuring 7.5 × 7 cm in diameter present in the right side of the colon, which infiltrated into the adjacent kidney, and swelling of the left supraclavicular and periaortic lymph nodes (Fig. 1). Therefore, she was referred to our hospital for further evaluation and treatment. At presentation, the patient was thin with a body mass index of 17.2 kg/m^2^ (height 176 cm and body weight 53.2 kg). Physical examination revealed that the eyelid conjunctiva was pale, and a palpable mass with poor mobility was present in the upper right abdomen. Laboratory tests revealed anemia (hemoglobin level 7.2 g/dL and hematocrit value 22.7%) and inflammation (white blood cell count 13,800/µL and C-reactive protein 6.534 mg/dL). The levels of tumor markers were within the normal range (cancer embryonic antigen 1.2 ng/mL and carbohydrate antigen 19–9 < 4.0 U/mL). Although colonoscopy revealed severe stenosis and edematous mucosa in the right colon that the endoscopic fiber could not pass through, no obvious neoplastic lesions were detected (Fig. 2).

A laparoscopic examination was performed under a clinical diagnosis of suspected right-sided colon cancer (clinical stage IV), which revealed no obvious peritoneal dissemination, and intraoperative cytological examination of the ascitic fluid was negative. Subsequently, laparotomy was performed to evaluate whether the tumor was resectable. When the omental bursa was opened, it showed that the tumor directly invaded the head of the pancreas, right kidney, and the second portion of the duodenum. In addition, direct invasion of the superior mesenteric vessels was observed. Therefore, radical resection of the tumor was considered impossible. A part of the tumor was resected and submitted for an intraoperative consultation, leading to the histopathological diagnosis of a malignant epithelioid tumor, unlike a typical colon cancer. Ileocecal vascularized bulky lymph nodes (No. 201 lymph node) with suspected metastases were sampled, and gastrojejunostomy with Braun’s anastomosis and ileotransversostomy were performed as palliative procedures. The surgery lasted 164 min, and the total blood loss was 13 mL.

**Fig. 3:**
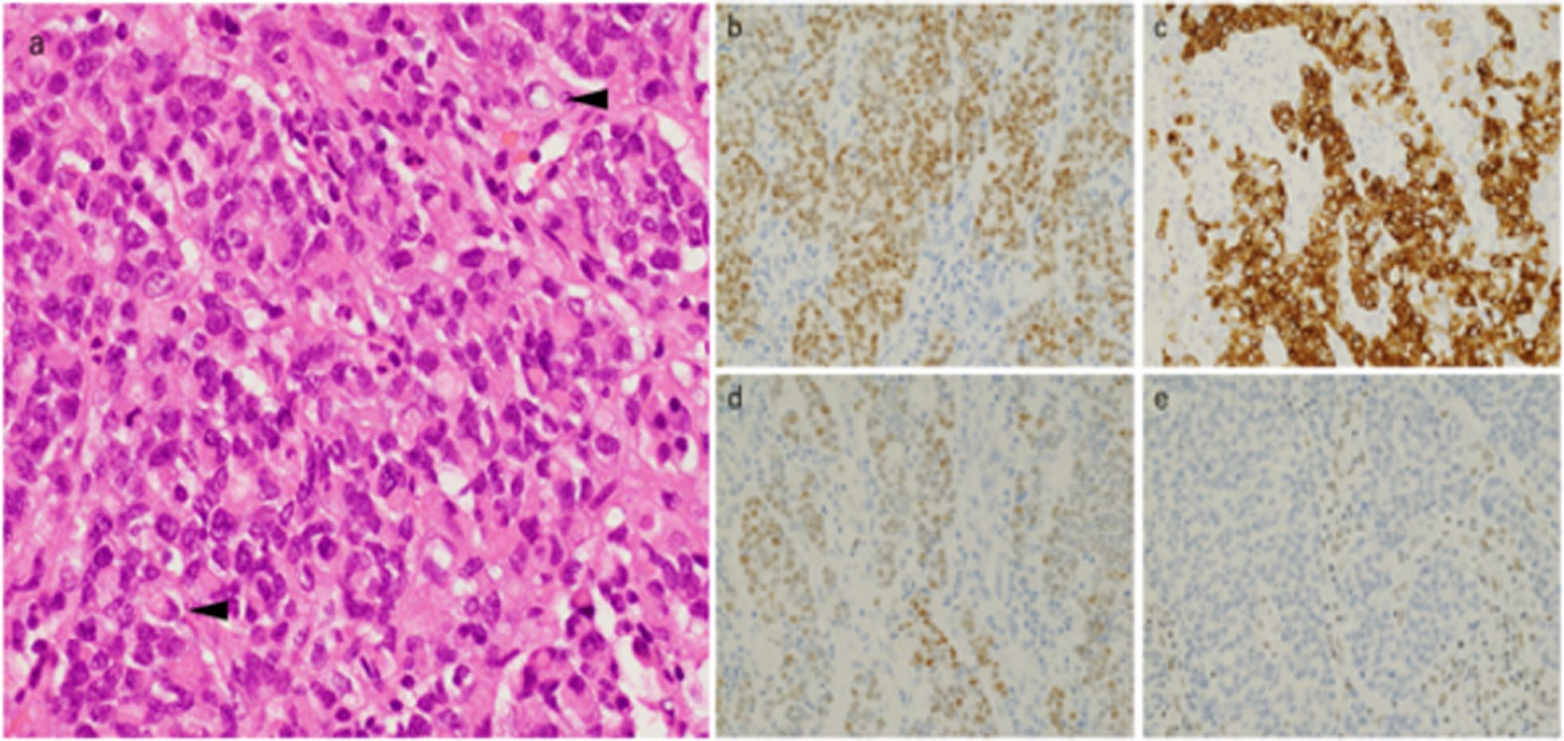
Histopathological and immunohistochemical findings of the lymph node. **a** The proliferation of the polygonal neoplastic cells having rich eosinophilic cytoplasm and eccentrically located large round-to-oval nuclei containing nucleoli (hematoxylin and eosin, × 200). The neoplastic cells express CDX-2 (**b**), keratin 20 (**c**), and HNF4alpha (**d**) (× 200). **e** Loss of *SMARCB1* expression is observed in the neoplastic cells (note: *SMARCB1* expression is noted in the non-neoplastic cells) (× 200)

Histopathological examination of the resected No. 201 lymph node demonstrated that the lymph node was occupied by sheets or irregular nests of neoplastic cells with geographic necrosis. These neoplastic cells were polygonal in shape with a rich eosinophilic cytoplasm and eccentrically located large round-to-oval nuclei containing conspicuous nucleoli, which are the characteristic features of rhabdoid appearance (Fig. 3a). Conventional tubular adenocarcinoma components were not observed. Immunohistochemical analyses revealed that these rhabdoid neoplastic cells were positive for keratin 20, CDX-2, and HNF4alpha (Fig. 3b–d) but negative for keratin 7, chromogranin A, and synaptophysin. These neoplastic cells showed a characteristic loss of INI1 (Fig. 3e). A metastatic *SMARCB1*-negative rhabdoid carcinoma in the lymph nodes was diagnosed.

The postoperative course was uneventful, and the patient resumed eating on the 3rd postoperative day. Two cycles of pembrolizumab 200 mg every 3 weeks were administered because molecular analysis of the metastatic carcinoma in the lymph node confirmed that the neoplastic cells were *RAS*-wild type, *BRAF*^V600E^-mutant, and MSI-high. However, no clinical benefit was noted, and the patient died 3 months postoperatively.

## Discussion

In the present report, we described the 6th case of *SMARCB1*-negative rhabdoid carcinoma occurring in the right colon. This is the first case of this type of tumor that was treated with pembrolizumab.

*SMARCB1* is a tumor suppressor gene that constitutes the SWItch/Sucrose Non-Fermentable (SWI/SNF) chromatin remodeling complex that causes a conformational change in the nucleosome, alters histone–DNA binding, and facilitates transcription factor access [9, 10]. Loss of *SMARCB1* is characteristic of malignant tumors with rhabdoid features. Some cases of rhabdoid carcinoma occurring in the gastrointestinal tract also showed the loss of this gene [1–3]. Fewer than 30 cases of colorectal rhabdoid carcinoma have been reported, although most were not analyzed for *SMARCB1* [8]. Only six cases, including the present case, showed loss of *SMARCB1* [1, 2, 6–8], and a few rhabdoid carcinoma cases with retained *SMARCB1* expression occurring in the colorectum have also been reported [11, 12]. In a study that analyzed 3,051 cases of colorectal carcinoma, *SMARCB1* loss was detected in only 0.46% of the cases. These tumors tended to have larger sizes, higher histological grading, and poorer prognosis [13]. Table 1 summarizes the clinicopathological features of previously reported cases of *SMARCB1*-negative rhabdoid carcinoma occurring in the colon, similar to the present case. The median age of the patients was 72 (range 41–81) years with male and female sexes being equally affected. All reported cases had a large tumor and metastases to lymph nodes and the liver. Most patients died of the disease within 6 months, although one case with *SMARCB1*-negative rhabdoid carcinoma in the cecum without tumor recurrence 48 months after surgery has been reported [7].

**Table 1:** Clinicopathological features of SMARCB1-negative rhabdoid carcinoma of the colorectum

Author	Age (years)	Sex	Location	Size (cm)	RAS/BRAF^V600E^ status	EGFR expression	SMARCAB1	MSI Status	Treatment	Metastasis	Outcome
Pancione et al. [6]	73	Female	Right-side colon	10 × 8	RAS wild/ BRAF^V600E^ mutation	Negative	Loss	High	Surgery, capecitabine, oxaliplatin	Liver	DOD 6 months
Agaimy et al. [1]	79	Male	Cecum	9 × 5 × 2	RAS wild/ BRAF^V600E^ mutation	NA	Loss	High	Surgery	LN	DOD 6 months
D'Amico et al. [7]	65	Male	Cecum	10 × 8 × 10	NA	NA	Loss	NA	Surgery, chemotherapy	LN	AWD 48 months
Tessier-Cloutier et al. [2]	81	Female	NA	NA	NA	NA	Loss	MLH1, MSH2, PMS2( +), MSH6(-)	NA	NA	NA
Kojima et al. [8]	41	Male	Sigmoid colon	7 × 6	RAS wild/ BRAF^V600E^ mutation	NA	Loss	Stable	Surgery, FOLFOX6, FOLFOXIRI	Liver, peritoneum, LN	DOD 2 months
Present Case	71	Female	Right-side colon	7.5 × 7	RAS wild/ BRAF^V600E^ mutation	NA	Loss	High	Pembrolizumab	LN	DOD 3 months

*NA* not available, *AWD* alive without disease, *DOD* died of disease, *LN* lymph node

The primary tumor was unresectable in the present case. The diagnosis of *SMARCB1*-negative rhabdoid carcinoma was histopathologically and immunohistochemically confirmed using a metastatic lymph node specimen. The neoplastic cells in the metastatic lymph nodes showed the colorectal phenotype because CDX-2 (intestinal marker) and HNF4alpha (gastrointestinal marker) were expressed. Keratin 7-negative and keratin 20-positive phenotypes were typical for colorectal carcinomas, although the loss of CDX-2 expression was noted in some colon rhabdoid carcinomas [6, 8]. Thus, we considered that the neoplastic cells in the metastatic lymph node were of right-sided colon origin, based on clinical and imaging test findings.

The deficiency of the mismatch repair system has been recognized in approximately 15% of colorectal carcinomas [14]. MLH1 epigenetic silencing is the most frequent event responsible for MSI. MSI-high status is characterized by a frequent right-sided colon location, poorly differentiated histology, signet ring cell carcinoma or mucinous carcinoma component, and numerous infiltrating lymphocytes [15]. *BRAF *^*V600E*^ mutation is also frequently noted in right-sided colon cancer, and patients with tumors with MSI-high status more commonly have this mutation [16]. Loss of MLH1 or MSH6 was also noted in *SMARCB1*-negative rhabdoid carcinoma of the colon (Table 1), and the present patient showed an MSI-high phenotype. Therefore, we administered two cycles of pembrolizumab because its efficacy was reported in patients with colorectal cancer with MSI-high status [17]. However, the patient died 3 months after the surgery without any clinical benefits. The most important factor in the resistance to ICIs therapy might be the high-grade aggressive rhabdoid tumor. In generally, the infiltration of CD8^+^ T cells into the tumor microenvironment is important for the efficacy of ICIs [18, 19]. Although details of CD8^+^ T cells in the tumor microenvironment were not available in this case because of the lack of the main tumor specimen. The use of ICIs is a promising therapeutic strategy, and therapeutic efficacy has already been reported in *SMARCB1*-negative sarcomas [20]. Additional clinical studies of ICI therapy for *SMARCB1*-negative colorectal rhabdoid carcinoma are required to establish a therapeutic strategy for this type of tumor.

Moreover, in some patients with metastatic colorectal cancer with the *BRAF *^*V600E*^ mutation, the combination of encorafenib, cetuximab, and binimetinib significantly prolonged overall survival and demonstrated a high response rate compared to the standard therapy [21]. Although it might be difficult to administer this triple therapy because of poor performance status, future combination therapy might be considered for treating rhabdoid colorectal cancer with *BRAF *^*V600E*^ mutation. In addition, the efficacy of EZH2 inhibitors in patients with advanced *SMARCB1*-negative epithelioid sarcoma has been reported [22]. Thus, colorectal rhabdoid carcinoma with loss of *SMARCB1* may benefit from these inhibitors. However, additional clinical studies are needed to clarify the therapeutic strategy for *SMARCB1*-negative rhabdoid colorectal carcinoma.

## Data Availability

All data generated or analyzed in the present study are included in this published article.

## Abbreviations

CDX-2: Caudal-type homeobox-2
HNF4alpha: Hepatocyte nuclear factor 4 alpha
ICI: Immune checkpoint inhibitor
INI1: Integrated interactor 1
MSI: Microsatellite instability
EZH2: Enhancer of zeste homolog 2; MLH1; MHS6
